# Intranasal Delivery of Targeted Nanoparticles Loaded With miR-132 to Brain for the Treatment of Neurodegenerative Diseases

**DOI:** 10.3389/fphar.2020.01165

**Published:** 2020-08-06

**Authors:** Yu Su, Bixi Sun, Xiaoshu Gao, Xinyue Dong, Lanbo Fu, Yingxin Zhang, Zhulin Li, Yue Wang, Hongyu Jiang, Bing Han

**Affiliations:** Department of Biopharmacy, School of Pharmaceutical Sciences, Jilin University, Changchun, China

**Keywords:** neurodegenerative diseases, MiR132, nose to brain delivery, nanoparticles, wheat germ agglutinin

## Abstract

Effective treatments for neurodegenerative diseases need to be developed. MiR132 is abundantly expressed in the brain, and it modulates neuron morphology and plays a key role in maintaining neuron survival. Regulating miR132 can effectively improve the symptoms of Alzheimer’s disease. It can also reduce cell death after cerebral hemorrhage, improve the microenvironment of hematoma lesions and provide a certain protective effect from brain damage after cerebral ischemia. MiR132 has great potential in the treatment of cerebral ischemia and Alzheimer’s disease. To prevent the decline of miR132 of miR132 levels in the blood, we used mouse and rat models of Alzheimer’s disease with ischemic brain injury, and then delivered Wheat germ agglutinin (WGA)-NPs-miR132 intranasally to treat neurological damage after cerebral ischemia. Synaptic protein expression levels in Alzheimer’s mouse models increased significantly after administration. We propose that, nasal delivery of WGA-NPs-miR132 is an interesting novel therapeutic approach for the treatment of neurodegenerative diseases.

**Graphical Abstract:**
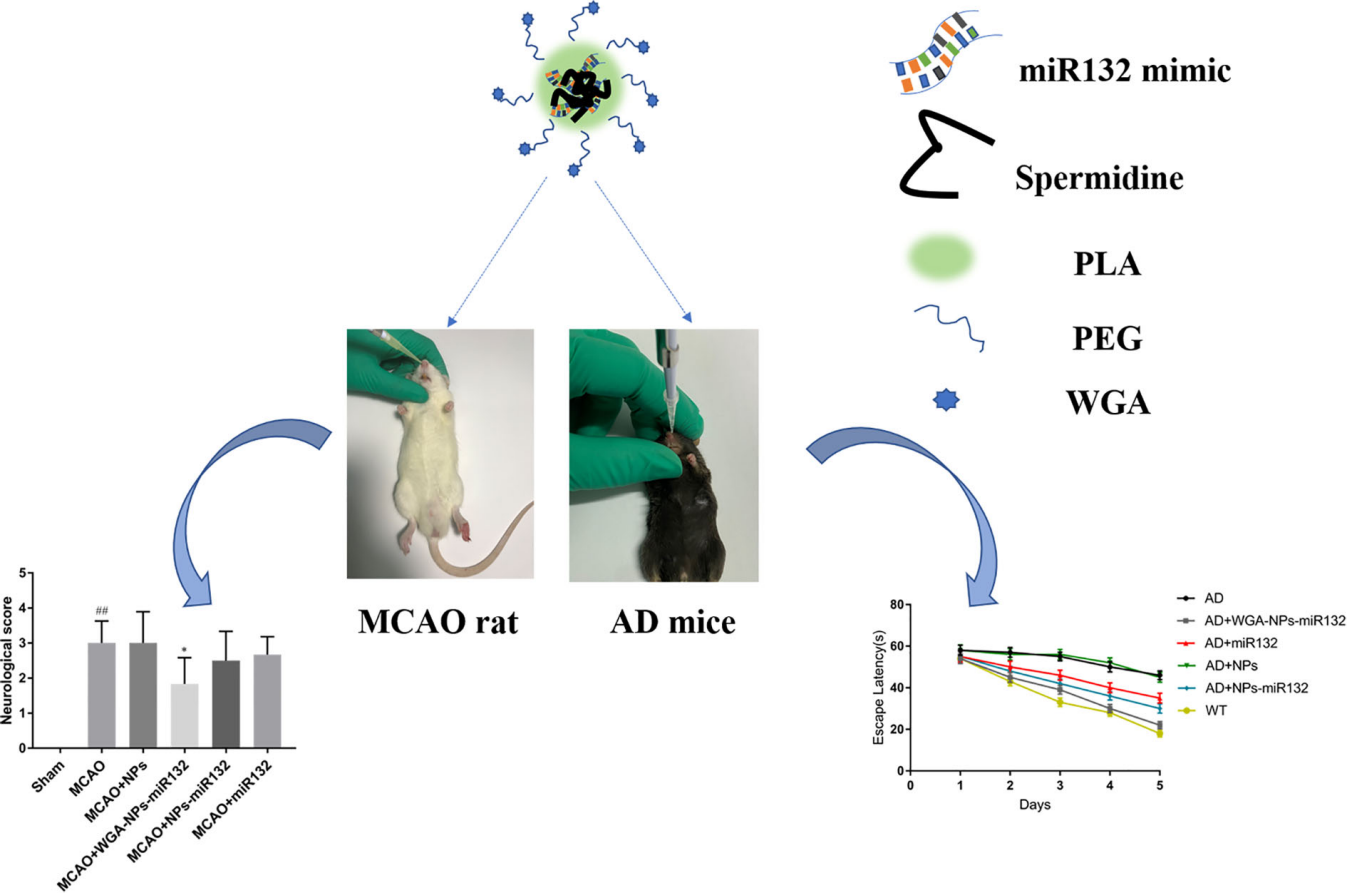


## Introduction

Although many drugs have been developed for treating central nervous system diseases, their therapeutic effects are still insufficient (Gitler et al., 2017). Gene therapy has attracted widespread attention in recent years, and studies have shown that many microRNAs in mammalian brains have dynamic functions in regulating biochemical pathways; the pathogenesis of neurodegenerative diseases can be associated with disrupted miRNA regulation, which may be caused by the disruption of a single miRNA or a miRNA family, so miRNA-based treatment of neurodegenerative disease has great potential (Karnati et al., 2015; Ferrante and Conti, 2017). miRNA mimics or inhibitors can be used to restore the normal function of specific miRNAs to achieve the purpose of neurodegenerative diseases (Pereira et al., 2017).

MiRNAs have been studied as treatments for diseases, but various therapeutic nucleic acid drugs cannot completely penetrate the blood–brain barrier (BBB) and enter brain tissues within an effective time window (Begley, 2004; Pardridge, 2005). Currently, there are two main types of methods used to deliver drugs to across the blood-brain barrier: invasive and noninvasive (Zhang et al., 2016). The invasive method, which is predominantly intracerebral injection, allows the drug to cross the blood–brain barrier and reach effective drug concentrations of the central nervous system. Its disadvantages are high cost and side effects such as infections and neuronal damage (Upadhyay, 2014). As a noninvasive treatment method for brain diseases, nasal administration has attracted increasing attention (Lochhead and Thorne, 2012; Meng et al., 2018).

Intranasal delivery primarily involves a drug entering the brain directly through the olfactory and trigeminal nerve pathways for therapeutic purposes; thus, the drug bypasses the blood–brain barrier and avoids the first-pass effects of the gastrointestinal tract and liver (Chen et al., 2016; Ahmad et al., 2017). Although nasal delivery has many advantages over other delivery methods, there are many degrading enzymes, such as cytochrome P450, aminopeptidase and aldehyde dehydrogenase, that inhibit the drug activity (Oliveira et al., 2016; Fan et al., 2018). Second, the nasal cilia ciliary clearance reduces the time of drug contact with nasal epithelial cells, and the retention time is short, so the absorption of drugs is limited (Merkus et al., 1998). Therefore, to enhance the stability of drugs in the nasal cavity and the ability of the nasal mucosa to absorb drugs, a delivery carrier is needed to protect the nucleic acid from degradation and resist nasal cilia clearance. As a potential platform for drug and gene delivery, nanoparticles have attracted great attention (Graff and Pollack, 2003). Due to their unique size, shape and surface characteristics, low biotoxicity and good biocompatibility, nanoparticles are able to protect therapeutic drugs from degradation by various degradants and enhance the concentrations of drug delivery. At the same time, in order to improve the uptake ability of nasal epithelial cells to nanoparticles and reduce the clearance rate of nasal cilia, further modification of nanoparticles is needed to enhance their delivery ability. Wheat germ agglutinin (WGA) has been reported to bind specific oligosaccharides in nasal epithelial cells, prolonging their residence time and promoting their internalization (Lehr, 2000; Shen et al., 2011). This selective affinity for the nasal mucosa is thought to enhance the delivery of WGA-modified nanoparticles from the nose to the brain (Liu et al., 2012). After intranasal administration, a WGA-NP enters the lamina propria through transcellularly, and then is transferred to the olfactory nerve bundle or the surrounding connective tissue. In addition, the surface of neurons in the brain also contains a large number of WGA receptors, so WGA can be effectively absorbed by neurons (Huh et al., 2010).

Although WGA-modified nanoparticle-encapsulated therapeutic drugs have been reported for nasal inhalation for the treatment of cerebral ischemia, WGA-modified nanoparticle-encapsulated miRNAs have not been reported for nasal delivery for the treatment of neurologically advanced diseases. Here, we will study the nasal administration of miRNAs coated with polymer nanoparticles modified by lectins and analyze their therapeutic effect on neurodegenerative diseases. Alzheimer’s disease (AD) and ischemic brain injury are two major and common neurodegenerative diseases. It has been shown that miR132 is abundantly expressed in the brain, and it regulates the morphology of neurons and plays a key role in maintaining neuronal survival (Scott et al., 2012). miR132 has protective effects on cerebral nerves during Alzheimer’s disease and following cerebral ischemia (Hernandez-Rapp et al., 2016; Zhang et al., 2017). We developed WGA-modified PEG-PLA nanoparticle with miR132 (WGA-NPs-miR132) for nasal delivery, and the neuroprotective effect of this method on AD model mice was evaluated by water maze tests and immunofluorescence analysis. The neuroprotective effect of the delivery method on the MCAO rat model was evaluated by quantification of infarct area, neuronal death and neuroinflammatory axis. The results indicated that miR132 inhaled through the nose had great potential for neuroprotection in Alzheimer’s disease and cerebral ischemia.

## Materials and Methods

### Materials

Maleimide-poly(ethylene glycol)3000-poly(lactic acid)70000(Male-PEG-PLA) and methoxy poly(ethylene glycol)3000-poly(lactic acid)50000 (MePEG-PLA) were purchased from Changchun Kemao Biological Technology Co., Ltd; 2-IT (2-iminothiolane hydrochloride), spermidine, DiR (1,1′-dioctadecyl-3,3,3′,3′-tetramethylindotricarbo-cyanine iodide), were purchased from Sigma-Aldrich (MO, USA). WGA (wheat germ agglutinin; MW 36000) was purchased from Changchun Xinjinji Biological Technology Co., Ltd; MiR132 mimic was purchase from Shanghai GenePharma Co., Ltd.

### Animals

APP/PS1 double transgenic mice (AD mouse model) used in this study were transgenic, carrying the human APP(APPswe) gene and a mutated human PS1 gene; Wild-type C57BL/6 mice were obtained from Beijing Zhishan Co., Ltd. [SCXK, (Jing) 2016-0007]. Male Sprague-Dawley rats (SD) (weighing 220–250 g) were purchased from Liaoning Changsheng Biotechnology Co., Ltd. The mice were 6-month-old (20–25g) and rats were bred under conditions including a 12 h light/dark cycle (temperature 23–25°C; relative humidity 50–60%) and free access to water and food. The experimental program was approved by the Ethics Committee of Jilin University. All animal studies were conducted in strict accordance with the approval by the Animal Ethics Committee of the School of Pharmaceutical Sciences, Jilin University. All efforts were made to minimize the number of animals used and their suffering.

### Preparation and Characterization of Nanoparticles

The w/o/w method was used to prepare PEG-PLA nanoparticles loaded with miR132. Briefly, miR132 and spermidine were mixed in enzyme-free water (N/P ratio 10:1) and stored at room temperature for 15 min to form a spermidine/miR132 complex. A total of 25 mg of Male-PEG-PLA and mPEG-PLA (1: 9, w: w) were dissolved in 1 ml of dichloromethane (DCM), and then the spermidine/miR132 complex was added dropwise to the DCM mix. A probe was used to sonicate the mix for 60 s in a water bath in an ice solution to emulsify it. The primary emulsion was added to 20 ml of a 2.5% PVA (w/v) aqueous solution and then was stirred, and the organic solvent was removed by rotary evaporation (Han et al., 2010; Shen et al., 2011). The nanoparticles were concentrated by centrifugation at 21,000 × g for 45 min and washed three-times with deionized water. WGA was thiolated by 1-h reaction with 2-iminothiolane (1:60 molar ratio). Nanoparticles and 2-iminothiocyclopentanethiolated WGA were incubated for 8 h at a molar ratio of WGA-maleimide of 1:3. WGA-NPs were purified by centrifugation at 21,000 × g for 45 min and then washed 3 times with 1 ml of PBS. MiRNA-free blank nanoparticles were prepared as follows. DiR-labeled nanoparticles were generated by adding DiR to the oil phase, and the preparation process was the same as above. The particle size and zeta potential of the nanoparticles were measured using the Malvern Nano-ZS90 dynamic light scattering particle size analyzer. Nanoparticles were imaged using a transmission electron microscope(TEM)(JEM-2100F). Use the Quant-iT™ RiboGreen^®^ RNA Assay Kit to determine the efficiency of miRNA loading. Follow the kit instructions.

### Biodistribution

Compare the biodistribution of WGA modified PEG-PLA nanoparticles and non-WGA modified PEG-PLA nanoparticles in rats. DiR-labeled NPs and DiR-labeled WGA-NPs were administered intranasally to AD mice or MCAO rat. Four hours later, experimental animals were perfused with physiological saline, and their brains and peripheral organs were collected. Fluorescence images of the brain and peripheral organs were obtained and analyzed using a bioluminescent imaging system (IVIS SPECTRUM, USA).

### Treatment of APP/PS1 Mice With WGA-NPs-miR132

APP/PS1 mice were randomly divided into 5 groups: AD, AD + WGA-NPs-miR132, AD+miR132, AD + NPs, AD + NPs-miR132, and C57/BL mice (which were the WT group) (n=6). Administered 20μL of nanoparticles by pipette, 5μL per nostril each time, alternating between each nostril every 2–3 min (miR132 simulated 40 pmol) (Salta et al., 2016). WT mice received an equal volume of a vehicle. Treatment was performed every other day for a total of 30 days. Then, after training and testing on the MWM maze, all the mice were sacrificed separately and brain tissue samples were taken for subsequent experiments.

### Morris Water Maze

APP/PS1 mice were randomly divided into 5 groups: AD, AD + NPs, AD + NPs-miR132, AD + WGA-NPs-miR132, and C57/BL mice (which were the normal group). The Morris water maze (MWM) test [21] measured improvements in spatial learning and memory formation ability in mice. A circular stainless steel container (150 cm in diameter, 60 cm in height) was divided into four quadrants. A circular platform (9 cm in diameter, 27 cm in height) was placed in the center of the target quadrant. Water was added to the pool so that the water level was 1 cm higher than the platform, and the water temperature was maintained at 22 ± 1°C. Milk powder was added to the water and mixed, so that the platform was covered. The incubation period occurred before the mice climbed onto the platform. If they could not find the escape platform within 60 s, the experimenters guided the mice to the platform and allowed them to stay there for 20 s. All mice were subjected to MWM tests four times per day at different times for five consecutive days. On the sixth day, space exploration experiments were conducted. An animal display video capture device with a connection display system was installed directly above the pool.

### ELISA for Aβ

ELISA kits were used to measure the levels of Aβ40 and Aβ42. Following the manufacturer’s protocol (Invitrogen, Carlsbad, CA, USA), an average of two replicate wells was used per sample. Quantitative analysis was performed by the external standard method, and a standard curve with a correlation coefficient > 0.99 was obtained.

### MCAO Model

SD rats were treated with MCAO as previously reported [22]. In short, rats were anesthetized with chloral hydrate and the left common carotid artery, external carotid artery (ECA) and internal carotid artery (ICA), and the vagus nerve were exposed and carefully separated. A 4–0 monofilament nylon suture (18.5–19.5 mm in length) was pushed from the ECA into the internal carotid lumen to block the MCA of the middle cerebral artery. After 120 min, reperfusion was performed by retracting the monofilament.

### Treatment of MCAO Rats With WGA-NPs-miR132

MCAO rats were randomly divided into 5 groups: MCAO, MCAO + NPs, MCAO + NPs-miR132, MCAO + WGA-NPs-miR132, and SD rats (which were the normal group) (n=15). Administered 20μL of nanoparticles by pipette, 5μL per nostril each time, which alternated between nostrils every 2–3 min (miR132 simulated 1pmol/g) (Zhao et al., 2018). Control rats received the same dose of a vehicle. Treatment was performed every other day for a total of 10 days.

### Neurological Score

Neurological deficits were evaluated with the Longa scoring system 12 days after reperfusion (Longa et al., 1989). The neurological score ranged from 0 to 4, with higher scores indicating more severe neurological deficits. The researchers who assigned the scores were blinded to the treatment groups of the animals. The scoring was used as follows: 0 and 4 points of animals were excluded, and 1–3 points of rats were included in the statistical criteria. All rats were then sacrificed separately, and brain tissue samples were taken for subsequent experiments.

### Brain Infarct Size Measurement

After neurological score assessment, coronal sections of the brain were collected and placed in a cuvette containing a 2% solution of 2,3,5-triphenyltetrazolium chloride (TTC) in the dark for 20 min at 37°C, and then the sections were fixed in 4% paraformaldehyde. Pictures were then captured with a camera. Infarct volume and total brain volume were quantitatively analyzed by an ImageJ analysis system. The cerebral infarct volume was expressed as a percentage of infarcted tissue relative to total brain tissue.

### Immunofluorescence Analysis

Paraffin sections were dewaxed and washed with PBS. After heating in a microwave for antigen retrieval, sections were washed with PBS.A blocking solution was added and was followed by incubation at room temperature for 30 min. Then, the sections were incubated with a primary antibody for the detection of PSD-95 (1:100 dilution, Abcam, Cambridge),SYN (1:200 dilution, Abcam, Cambridge) at 4°C overnight. FITC/TRITC fluorescent secondary antibodies (goat anti-rabbit IgG H&L (Alexa Fluor^®^ 488), 1:500, Abcam, Cambridge) were added and incubated with the sections in the dark for 30 min. Then, the sections were washed 3 times with PBS, stained with DAPI, incubated for 5 min at room temperature, and washed 3 times with PBS. The sections were sealed and scanned by laser scanning confocal microscopy. Positive cells were analyzed and counted by laser scanning confocal microscopy using ImageJ image analysis software.

### Neuronal Apoptosis Analysis

The prepared paraffin sections were stained according to the instructions contained in a TUNEL assay kit (Roche Molecular Biochemicals, Inc., Mannheim, Germany) to evaluate the apoptosis of brain tissue in each group in the AD model and MCAO model. The nuclei were stained by adding DAPI dye. For the measurement to quantify a factor of cell death, we calculated the ratio of apoptotic cells to nuclei as apoptotic cells (%).

### Real-Time Fluorescent Quantitative RT-PCR

The brain tissue of mice and rats were obtained after 24 h of reperfusion, and total RNA was extracted from the brain using TRIzol reagent (Invitrogen, New York, USA). cDNA was synthesized by using a PrimeScriptTM reverse transcription kit. The primer sequence of miR132:5’—CCAGCATAACAGTCTACAGCCA— 3’ (forward); 5’—TATGGTTGTTCACGACTCCTTCAC— 3’ (reverse); U6: 5’—CTCGCTTCGGCAGCACA— 3’(forward); 5’— AACGCT TCACGAAT T TGCGT— 3’(reverse).

### Western Blot Analysis

An appropriate amount of RIPA lysate buffer (Beyotime, Nanjing, Jiangsu, China) was added to extract the protein from tissues for processing. Protein concentration was calculated by BCA assay (Beyotime, Nanjing, Jiangsu, China). Each sample was resolved by 8% SDS-PAGE and then was transferred to a nitrocellulose membrane. The membrane was then blocked with 5% skim milk for 2 h before being washed for 5 min. Membranes were incubated at 4°C with primary antibodies against SYN (Abcam, Cambridge, MA, USA; 1:500), PSD‐95 (Abcam, Cambridge, MA, USA; 1:500), GAPDH (Abcam, Cambridge, MA, USA; 1:5000), Iba-1 (1:1000 dilution; Abcam, Cambridge, UK). GFAP (Abcam, Cambridge, MA, USA;1:1000);β-actin (Abcam, Cambridge, MA,1:1000). A horseradish peroxidase-labeled secondary antibody (1:2000) was then added. After washing, the membrane was developed using chemiluminescence, and then it was exposed and photographed. The acquired images were analyzed using ImageJ image processing software to quantify the intensity of the bands.

### Statistical Analysis

The analysis of the data was performed using GraphPad Prism software. One-way ANOVA followed by Tukey’s test was used for multiple comparisons. A value of P<0.05 was considered to indicate statistical significance.

## Result

### Characterization of Nanoparticles

**Figure 1:**
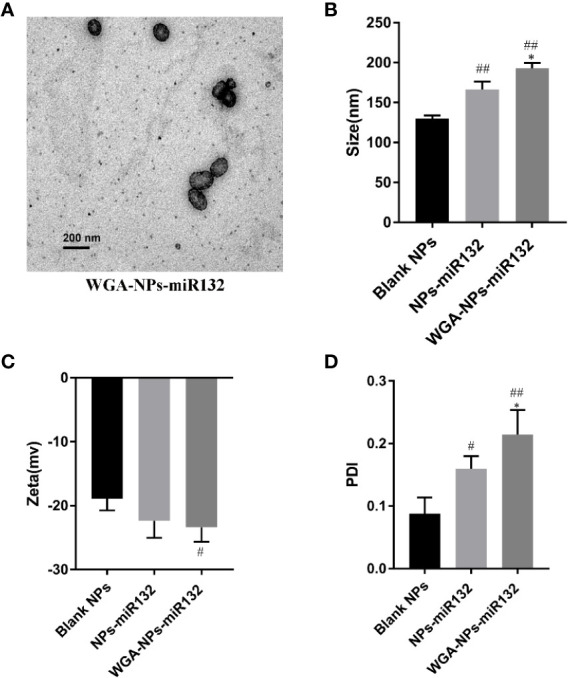
Characterization by **(A)** transmission electron microscopy, **(B)** size, **(C)** zeta potential and the PDI **(D)**. ^#^P < 0.05, ^##^P < 0.01 compared with the Blank NPs; *P < 0.05, compared with the NPs-miR132.

Transmission electron microscopy (TEM) images showed that the prepared nanoparticles were regular spheres. We also measured the size and zeta potential of the nanoparticles using a Malvern particle size analyzer. The average particle size of WGA-NPs-miR132 was approximately 191 nm, and the obtained polydispersity index (PDI) was less than 0.25 (**Figure 1**). The nanoparticles were stable in aqueous solution and had good homogeneity. The WGA conjugation did not significantly change the surface charge of NP. The entrapment efficiency (75.6 ± 11.25%) of miR132 loaded in nanoparticles was calculated using Quant-iT™ RiboGreen^®^ RNA Assay Kit.

### Distribution of Nanoparticles in the Mice

**Figure 2:**
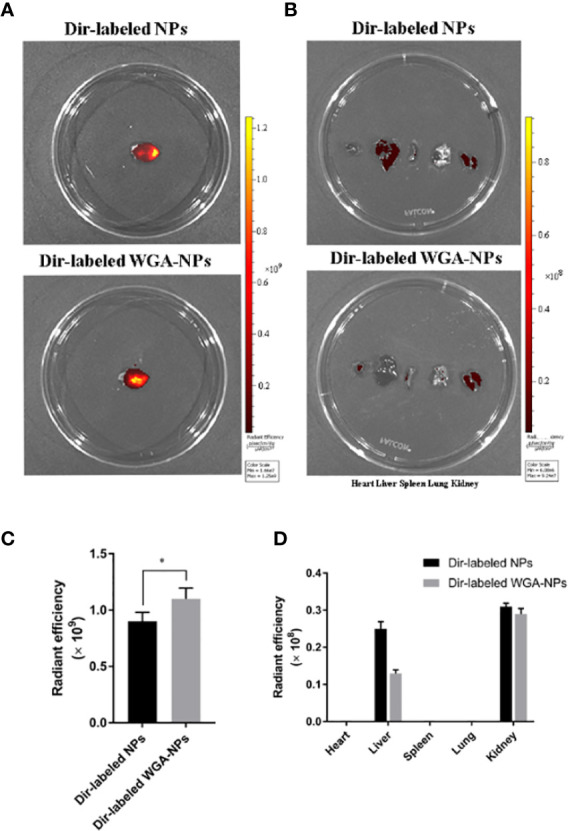
Biodistribution of DiR-labeled NPs and DiR-labeled WGA-NPs after intranasal administration in the AD model. Four hours after intranasal administration, the combination of WGA with nanoparticles caused enhanced accumulation of the nanoparticles in the brain (n = 3). **(A)** Biodistribution of DiR-labeled NPs and DiR WGA-NPs in brain. **(B)** Ex vivo fluorescence imaging of major organs. **(C)** Quantification of DiR-labeled NPs and DiR WGA-NPs accumulated in brain. **(D)** Quantification of DiR-labeled NPs and DiR WGA-NPs distributed in major organs excised from the mice. ^*^p < 0.05; DiR-labeled WGA-NPs vs. DiR-labeled NPs.

APP/PS1 mice were intranasally administered DiR-labeled nanoparticles, and the biodistribution of nanoparticles in experimental animal models was observed with a small animal fluorescence imaging system. After 4 h, the accumulation of WGA-NPs in the brain was significantly higher than that of unmodified NPs, which were mainly distributed in the liver, kidney, and spleen (**Figure 2**).

### Intranasal Delivery of miR132 Improves Cognitive Ability in APP/PS1 Mice

**Figure 3:**
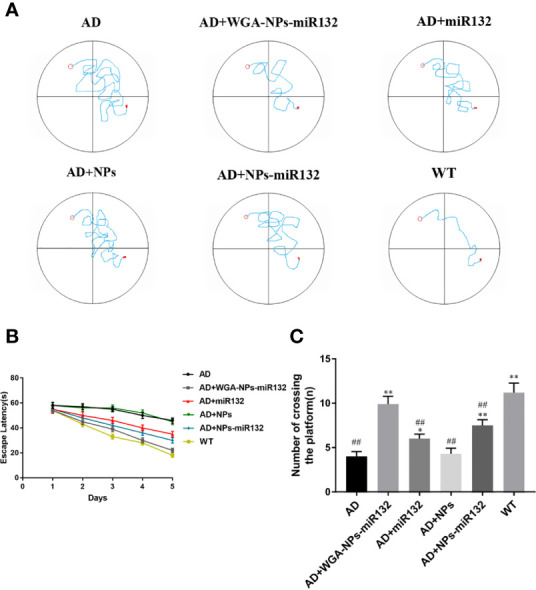
Morris water maze test. **(A)** Representative trajectory of each group in place navigation test. **(B)** The escape latency of the six groups in place navigation test. **(C)** The times crossing the target quadrant. ^##^P < 0.01 compared with the WT group; ^*^P < 0.05, ^**^P < 0.01 compared with the AD group (n = 6).

The Morris water maze was used to conduct behavioral tests on APP/PS1 mice to assess their spatial learning ability over time by observing them climb onto a platform to escape water (**Figure 3**). During the first two days of the test period, no significant differences were observed among the groups. On the third day, the AD group of mice escaped the incubation period. Compared with the AD group, the escape latency time of the AD+ WGA-NPs-miR132 group and AD+ NPs-miR132 group was significantly decreased. Compared with the AD group, the treatment effect of the miR132 group is milder (**Figure 3B**). In the probe experiment, we observed and recorded the number of times the mice passed over the platform. Among the groups, the amount of time that the AD+ WGA-NPs-miR132 group and AD + NPs-miR132 group spent passing over the platform significantly decreased. The results of MWM analysis showed that the intranasal delivery of WGA-NPs-miR132 improved the learning and memory function of APP/PS1 mice.

### WGA-NPs-miR132 Effectively Attenuated the Aβ Levels in APP/PS1 AD Mice

**Figure 4:**
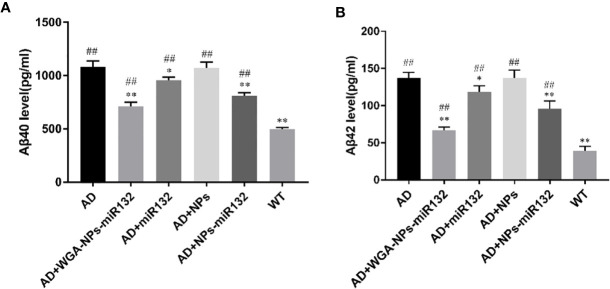
Changes in hippocampal amyloid-β (Aβ) levels in APP/PS1 mice. **(A)** ELISA of Aβ40 in the hippocampus of each group. **(B)** ELISA of Aβ42 in the hippocampus of each group. ^##^P < 0.01 compared with the WT group; ^*^P < 0.05, ^**^P < 0.01 compared with the AD group (n=3).

Aβ levels were upregulated early in the progression of AD, and this change was strongly correlated with a decline in cognitive function (Pan et al., 2018). Studies have shown that knockout of the miR132 gene in AD transgenic mice results in impaired memory and increased Aβ expression (Min et al., 2010). We used ELISAs to measure hippocampal Aβ40 and Aβ42 levels and evaluate the use of nasal delivery of WGA-NPs-miR132 in Alzheimer’s disease. The results showed that the levels of Aβ40 and Aβ42 in the AD + WGA-NPs-miR132 and AD + NPs-miR132 groups were significantly lower than those in the AD group (**Figure 4**). The treatment effect of AD + WGA-NPs-miR132 was significantly higher than that of the AD + NPs-miR132 group. There was no significant difference between the WGA-NPs and AD groups. The data showed that miR132 could partially improve cognitive functions of mice, but the effect of nasal aspiration delivery of naked-miR132 was not significant. This may be because miRNAs were less stable in the nasal mucosa or were easily cleared by the nasal mucosa and could not efficiently reach the target site.

### WGA-NPs-miR132 Reduces Neuronal Death in AD Mice

**Figure 5:**
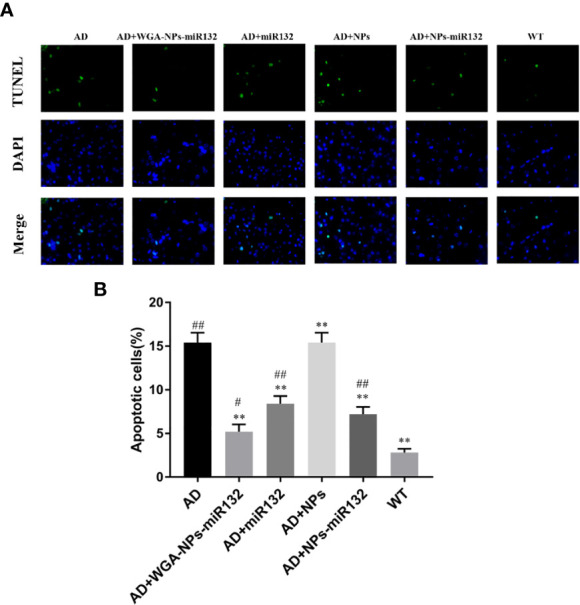
TUNEL assay results. Brain sections of mice were subjected to TUNEL staining to measure cell death. **(A)** Images of TUNEL staining in rat brain cortex. **(B)** The number of TUNEL-positive cells is presented. ^#^P < 0.05, ^##^P < 0.01 compared with the WT group; ^**^P < 0.01 compared with the AD group (n = 3).

We evaluated cell death in six experimental groups. Brain tissue sections from each group were subjected to TUNEL staining. The experimental results showed that compared with the normal group, the number of TUNEL-positive cells in the AD and AD + WGA-NPs groups increased, and a large number of neurons were apoptotic. The staining of TUNEL-positive cells in the AD + WGA-NPs-miR132 and AD + NPs-miR132 groups was significantly lower than that in the AD group (**Figure 5**).

### MiR132 Expression in Mouse Brain After Nasal Delivery of WGA-NPs-miR132

**Figure 6:**
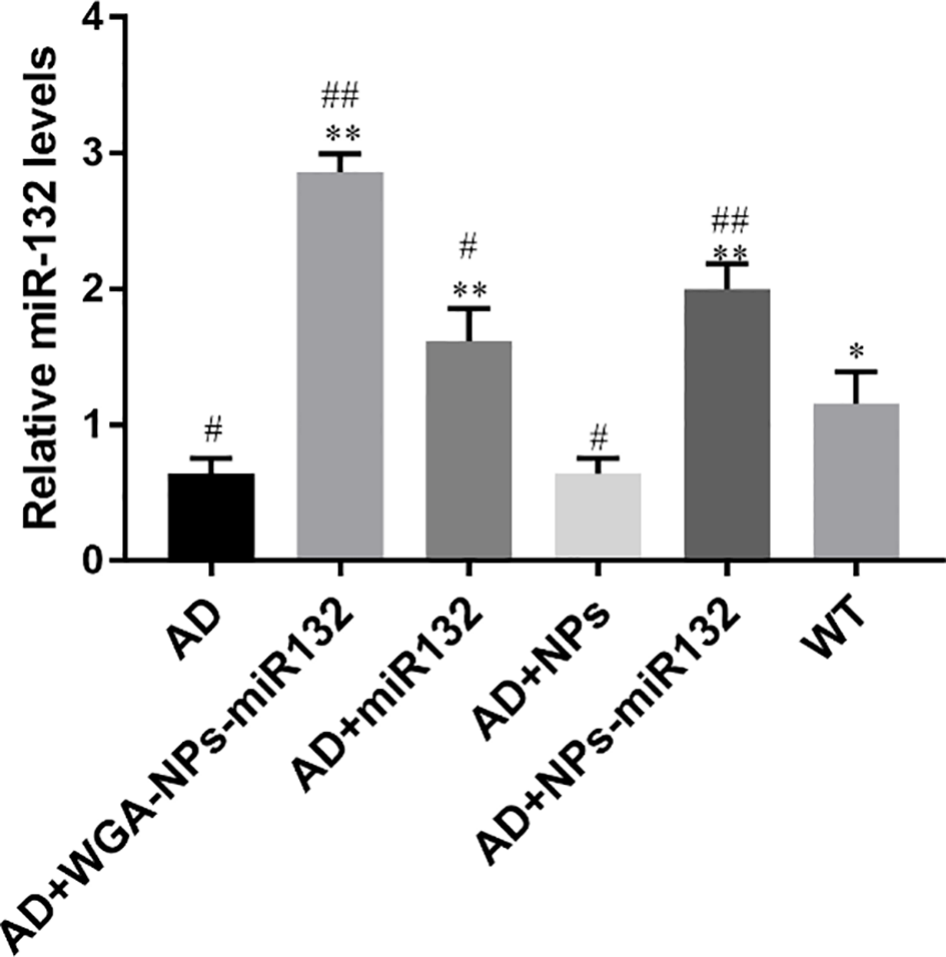
MiR132 was highly expressed in the temporal cortex after miR132 was nasally delivered. ^#^P < 0.05, ^##^P < 0.01 compared with the WT group; ^*^P < 0.05, ^**^P < 0.01 compared with the AD group (n = 3).

MiR132 in the brain parenchyma was detected by real-time PCR, and it was found to be significantly increased in the WGA-NPs-miR132 group (**Figure 6**).

### PSD95 and SYN Expression Levels in Mice Were Detected by Immunofluorescence

**Figure 7:**
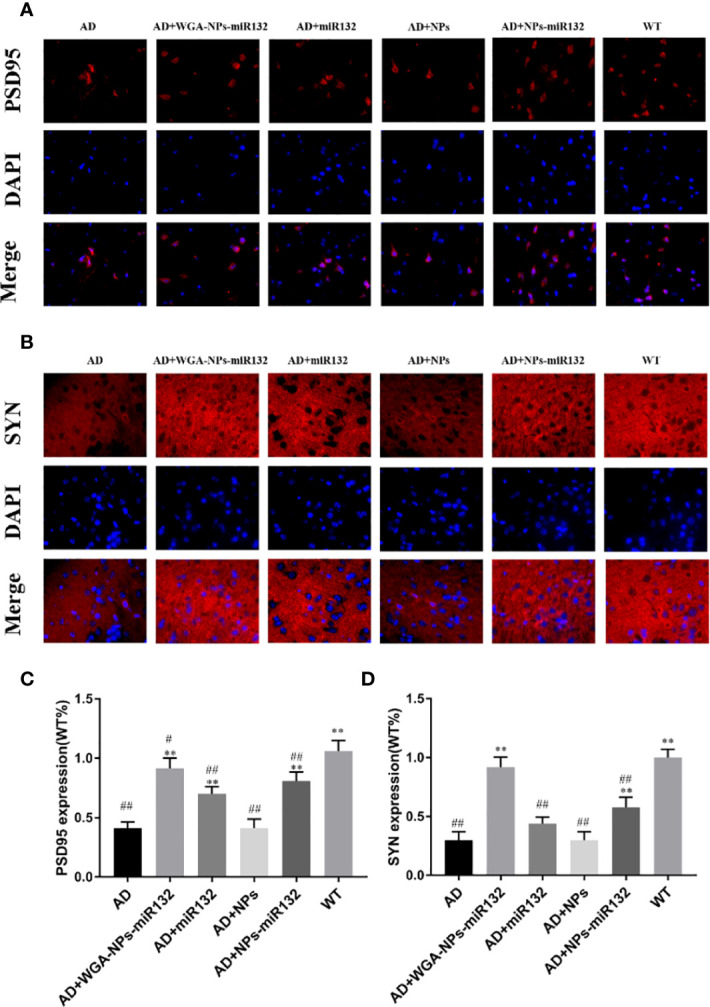
Immunofluorescence was used to detect the expression of PSD95 and SYN in the cerebral cortex. Representative images depicting the immunolabeling of PSD95 **(A)** and SYN **(B)** in the cerebral cortex and quantitative analysis of PSD-95 **(C)** and SYN **(D)** reveals their expression in the mouse cerebral cortex. ^#^P < 0.05, ^##^P < 0.01 compared with the WT group; ^**^P < 0.01 compared with the AD group (n = 3).

Synaptic plasticity defects are increasingly recognized as a factor affecting AD pathogenesis. PSD-95 is the main scaffold protein in excitatory synapses and postsynaptic densities, and has been characterized as one of the most abundant scaffold proteins in excitatory neurons, where it plays a key role in synaptic plasticity. Located in the presynaptic membrane, SYN mainly regulates the release of neurotransmitters (Jin et al., 2019). PSD95 and SYN were observed by immunofluorescence on the cell membrane (**Figures 7A, B**). Quantitative analysis showed that the PSD95 and SYN fluorescence intensity of the AD + WGA-NPs-miR132 group and AD + NPs-miR132 group was significantly higher than that of the AD model group. Compared with the WGA + NPs-miR132 group, the miR132 group had a lower fluorescence intensity, primarily due to the high affinity interaction of the WGA conjugated to the nanoparticle surface with the neuron surface receptors and the nanoparticle protects miR132 from being degraded and can reach the target site through nasal cavity (**Figures 7C, D**).

### PSD95 and SYN Expression Levels in Mice Were Detected by Western Blot

**Figure 8:**
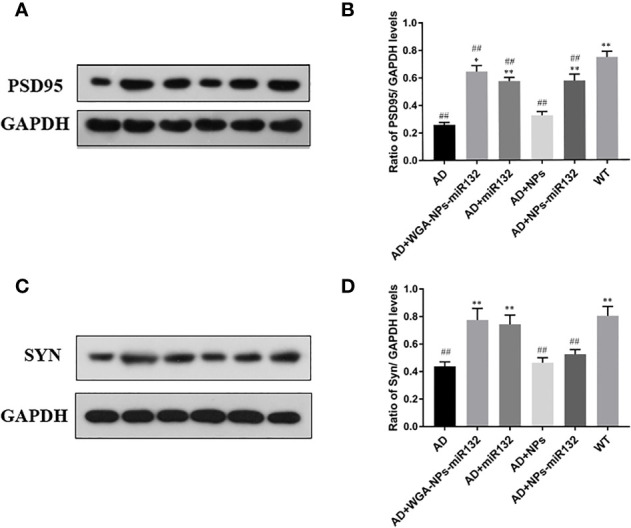
Analysis of PSD95 and Syn expression in the cerebral cortex by Western blotting **(A**, **C)**. Data represent the mean ± SD of four independent experiments. Comparison of PSD95 protein levels among the six groups **(B)**; Comparison of Syn protein levels among the six groups **(D)**. ^##^P < 0.01 compared with the WT group; ^*^P < 0.05, ^**^P < 0.01 compared with the AD group (n = 3).

Changes in synaptic protein expression were detected by Western blot (**Figure 8**). The results showed that the expression of PSD 95 and SYN decreased in the AD group. Both the AD + WGA-NPs-miR132 group and the AD + NPs-miR132 group exhibited significantly increased expression of PSD 95 and SYN in the hippocampal cortex.

### Nanoparticle Biodistribution in Rats

**Figure 9:**
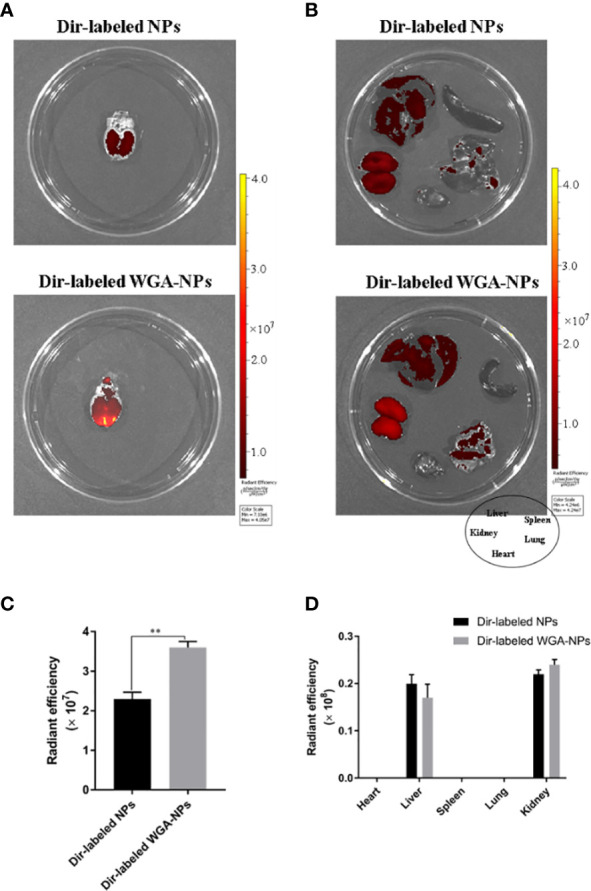
Biodistribution of WGA-NPs and WGA-NPs after intranasal administration in the MCAO model. Four hours after intranasal administration, the combination of WGA with nanoparticles enhanced the accumulation of nanoparticles in the brain. **(A)** Biodistribution of DiR-labeled NPs and WGA-NPs in brain from the rats. **(B)** In vitro fluorescence imaging of peripheral organs from the rats. **(C)** Quantification of DiR-labeled NPs and DiR WGA-NPs accumulated in brain from the rats. **(D)** Quantification of DiR-labeled NPs and DiR WGA-NPs distributed in peripheral organs excised from the rats. ^**^p < 0.01; DiR-labeled WGA-NPs vs. DiR-labeled NPs (n = 3).

DiR-labeled nanoparticles were administered to MCAO rats through the nasal cavity, and the biological distribution of nanoparticles in the MCAO model was observed by a small animal fluorescence imaging system. After 4 h, the accumulation of WGA-NP in the brain was significantly higher than that of unmodified NP, which was mainly distributed in the liver, kidney, and spleen. The appearance in the lung may be due to inhalation during administration (**Figure 9**).

### Neuroprotective Effect of WGA-NPs-miR132 After MCAO

**Figure 10:**
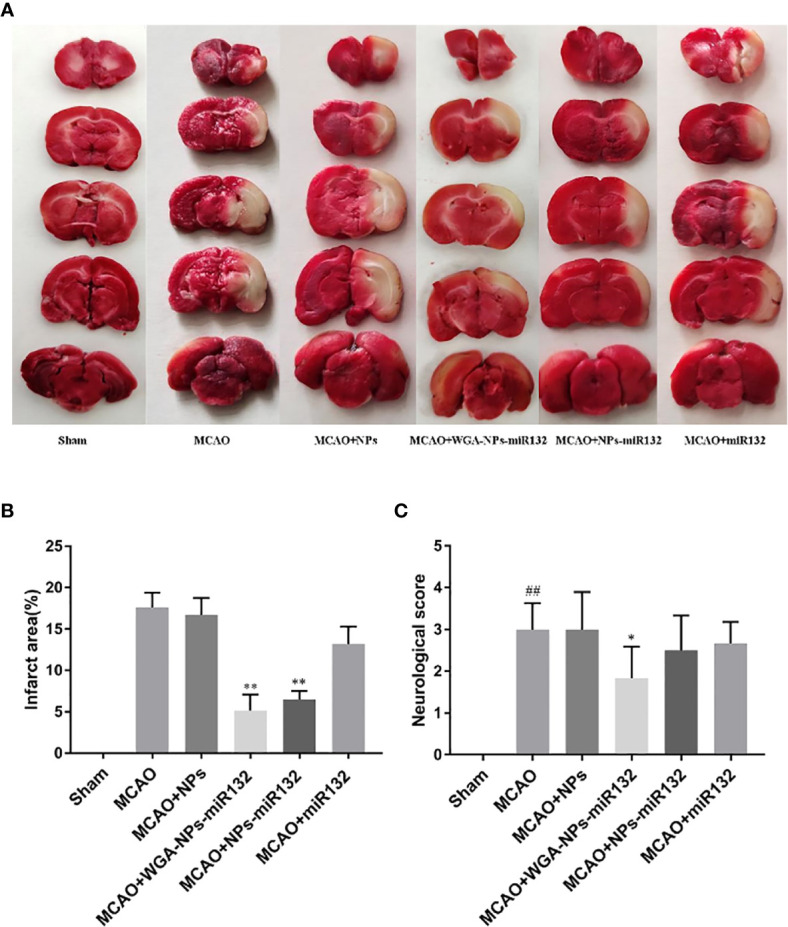
Neuroprotective effect of WGA-NPs-miR132 on nerve injury. **(A)** Representative images of TTC-stained brain sections from different groups in 24 h after MCAO. **(B)** Quantitative analysis of infarct volume in different groups (n = 3). **(C)** Neurological scores of different groups evaluated 24 h after MCAO (n = 8). ^##^P < 0.01 compared with the Sham group; ^*^P < 0.05, ^**^P < 0.01 compared with the MCAO group.

Neurological scores and TTC tests were used to verify the effect of nasal administration of WGA-NPs-miR132 on cerebral ischemia-reperfusion. TCC staining was used to assess the size of the cerebral infarction area; the lesion area of the cerebral infarction was not stained white. The MCAO treatment lasted 2 h, and brain infarct size and neurological score were measured at 24 h after reperfusion. As shown in **Figure 10**, the infarct area of the model group and the WGA-NPs group was basically the same, and the neurobehavioral score had not improved significantly, indicating that the nanoparticles themselves had no therapeutic effect; however, the administration of WGA-NPs-miR132 improved the neurobehavioral score and significantly reduced the infarct size. Naked-mir132 had a certain effect on the infarct area, but the effect was not ideal. This suggested that nanoparticles could protect miR132 from nasal cilia or related enzymes to a certain extent.

### WGA-NPs-miR132 Reduced Neuronal Death After MCAO

**Figure 11:**
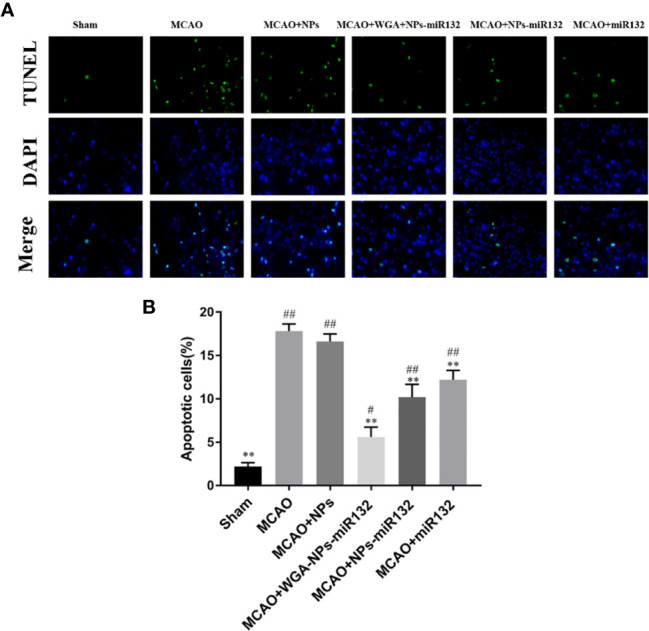
TUNEL staining results. Brain sections of rats underwent TUNEL staining to measure cell death. **(A)** Images of TUNEL staining in rat brain cortex. **(B)** The number of TUNEL-positive cells. ^#^P < 0.05, ^##^P < 0.01 compared with the Sham group; ^**^P < 0.01 compared with the MCAO group (n = 3).

We evaluated cell death rates in each group after cerebral ischemia. Brain tissue sections from each group were stained with TUNEL (**Figure 11**). The experimental results showed that compared with the MCAO group, the number of TUNEL-positive cells and the number of apoptotic neurons in the MCAO + WGA-NPs and MCAO + miR132 groups were reduced.

### MiR132 Expression After Intranasal Delivery of WGA-NPs-miR132

**Figure 12:**
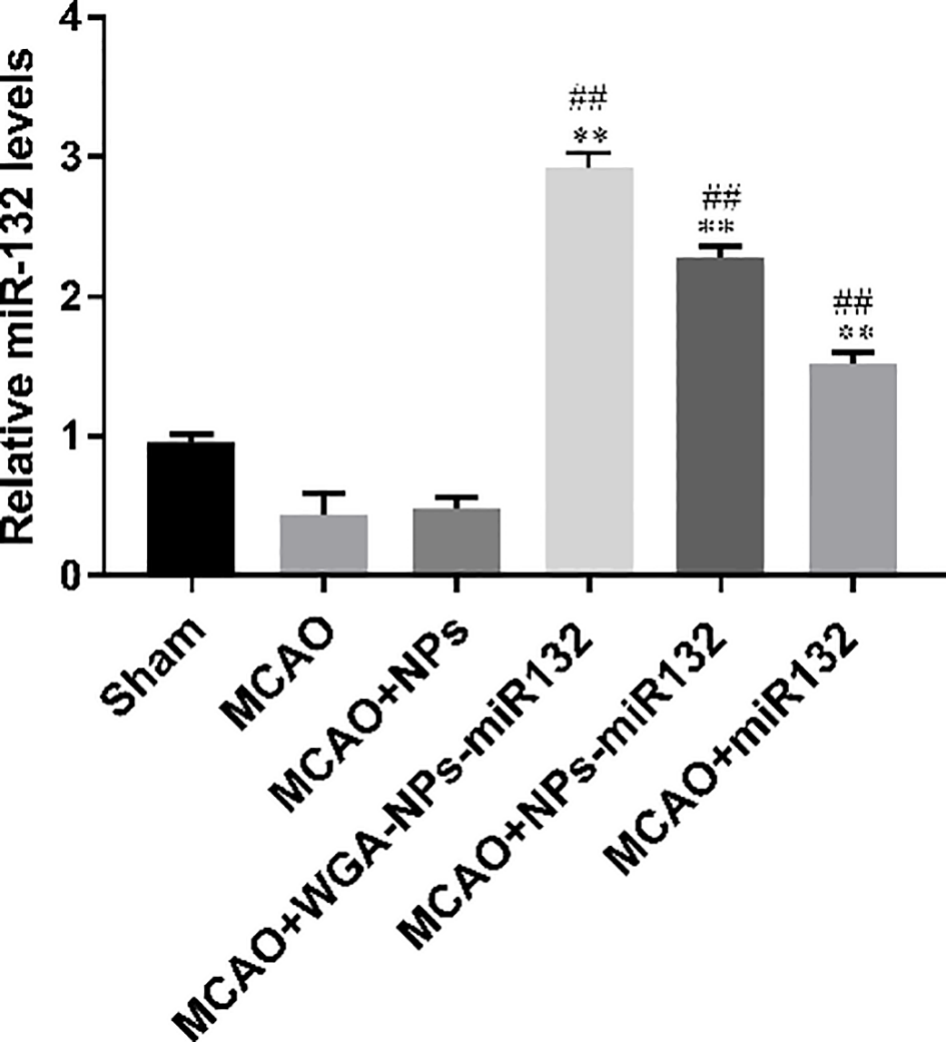
MiR132 was highly expressed in the temporal cortex after miRNA-132 was delivered nasally. ^##^P < 0.01 compared with the Sham group; ^**^P < 0.01 compared with the MCAO group (n = 3).

MiR132 Brain parenchyma was detected by real-time PCR of the brain tissue, which was significantly increased in the WGA-NPs-miR132 group (**Figure 12**).

### Western Blot Detection of Rat GFAP and Iba-1 Expression Levels

**Figure 13:**
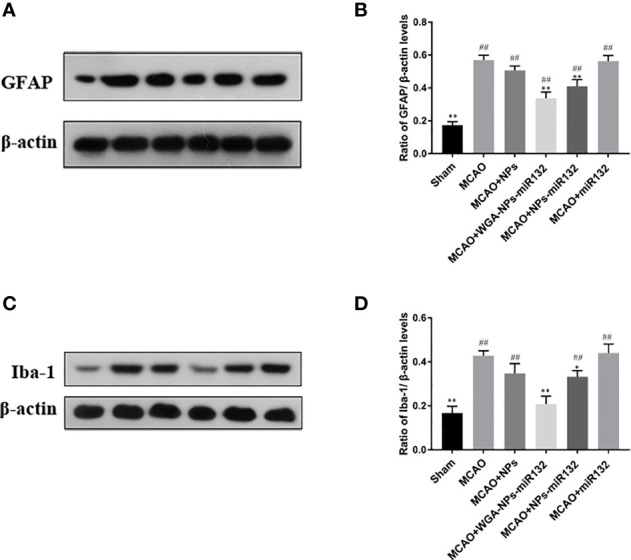
Analysis of the expression of GFAP and Iba-1 in the cerebral cortex, as assessed by Western blotting **(A, C)**. Data represent the mean ± SD of four independent experiments. Comparison of GFAP protein levels among six groups **(B)**; Comparison of Iba-1 protein levels among six groups **(D)**. ^##^P < 0.01 compared with the Sham group; ^*^P < 0.01, ^**^P < 0.01 compared with the MCAO group (n = 3).

Changes in synaptic protein expression were measured by western blot (**Figure 13**). The results showed that both the MCAO + WGA-NPs-miR132 group and the MCAO + NPs-miR132 group significantly reduced the expression of GFAP and Iba-1 in the hippocampal cortex, while there was little difference between the naked miR132 group and the AD group.

## Discussion

Neurodegenerative diseases are becoming increasingly common, and pose a great threat to human health. They are classified as acute and chronic neurodegenerative disease. The former mainly includes cerebral ischemia (CI), and brain injury (BI); the latter includes Alzheimer’s disease (AD), Parkinson’s disease (PD), Huntington’s disease (HD), and amyotrophic lateral sclerosis (ALS). The incidence of neurodegenerative diseases is increasing year by year, so we need to find quick and effective methods for preventing and treating these diseases (Gitler et al., 2017). MiRNAs not only participate in the development of various pathologies, such as cardiovascular disease, cataracts and cancer, but they also play an important role in various neurodegenerative diseases and have an important impact on the development of the central nervous system through the regulation of neurogenesis and synaptic functions (Silvestro et al., 2019). MiR132 is one of the most abundant miRNAs in the central nervous system, and plays a key role in neural development and regulation of neuronal activity (Edbauer et al., 2010). Previous studies have shown that miR132 regulation can effectively improve the symptoms of Alzheimer’s disease, and knocking out miR132 can increase the accumulation of soluble and insoluble amyloid Aβ (Ambegaokar and Jackson, 2012; Xu et al., 2019). Accumulation and deposition of Aβ in the brain can interfere with related signaling pathways, thereby causing changes in synaptic morphology and function and reducing synapses. Synaptic-associated proteins are an important part of maintaining synaptic morphology and function. Their loss can lead to deterioration of neurological functions, such as cognitive impairment. Studies have shown that miR132 can reduce cell death after cerebral hemorrhage, improve the microenvironment of hematoma lesions, protect the integrity of the blood-brain barrier, reduce cerebral edema and have provide a protective effect to prevent brain injury after cerebral ischemia (Zuo et al., 2019). MiR132 has potential application value in the treatment of Alzheimer’s disease and cerebral ischemia. However, due to the existence of the blood–brain barrier, the use of nucleic acid drugs to treat central nervous system diseases by oral or intravenous administration is limited, and the bioavailability is reduced. Direct intracerebral injection is inconvenient and can easily cause psychological and physical pain in patients.

In this study, we mainly focused on the therapeutic effect of miRNA delivery *via* nasal administration on neurodegenerative diseases. Nasal inhalation allows the drug to enter the central system directly through the trigeminal nerve and olfactory nerve without crossing the blood–brain barrier (Lochhead and Thorne, 2012). We selected miR132 as a neuroprotective agent for cerebral ischemia and improved memory in Alzheimer’s disease. However, without an effective *in vivo* modification of the miRNA, its transmission from the nose to the brain may be inhibited. Since naked miRNA molecules are water soluble and have a net negative charge, they are easily degraded or excreted in the mucosa after administration (Guo et al., 2016; Chen et al., 2018). Therefore, it is necessary to find a carrier with high safety, good stability and a specific targeting effect. Polylactic acid (PLA) is biocompatible and biodegradable. Copolymerization with other polymers [such as polyethylene glycol (PEG)] can produce mPEG-PLA copolymers with a core-shell structure in an aqueous environment. Such a structure has been widely used as a drug carrier in many studies. To improve the permeability of the cell membrane and reduce the removal of nanoparticles by nasal cilia, we conjugated lectins with PEG-PLA nanoparticles. Previous experiments have shown that WGA-NP is a safe and effective carrier for intranasal delivery to the central nervous system of the brain (Li et al., 2019). WGA binds to specific oligosaccharides in nasal epithelial cells. After nasal administration, WGA-NPs enter the lamina propria through cells, and then they are further transported into the brain through the olfactory pathway and trigeminal nerve pathway. The olfactory pathway has been reported to be a major component of nasal administration, delivering therapeutic drugs directly into the central nervous system. The continuous perineurial channels generated by the olfactory ensheathing cells that envelop the axons of olfactory cells are kept open. As a result, intranasally administered WGA-NPs may reach the brain *via* extracellular transport along olfactory nerves. When the particle size of WGA-NPs is less than 0.2μm, the nanoparticles may be transported along the trigeminal nerve through intracellular mechanisms (driven by axonal flow). The particle size distribution of nanoparticles was of great significance in determining mucosal penetration, drug release behavior, and prospected for nasal administration. Particles smaller than 1μm could pass through the nasal cavity, while particles larger than 10μm were deposited in front of the nose and had a smaller absorption area. Smaller particles tended to accumulate in the brain (Alex et al., 2016). Our prepared nanoparticles had an average size of 191 nm and could be inhaled through the nose to the treatment site. WGA-NPs prepared in our *in vitro* cytotoxicity test were shown to be safe and nontoxic and could be used as a miR132 intracerebral delivery vehicle.

We evaluated the treatment of Alzheimer’s disease with nasal inhalation delivery of WGA-NPs-miR132 through analysis of water maze experiments, Aβ, SYN, and PSD95 levels and apoptotic ratios, which were representative indicators of cognitive dysfunction. The water and maze tests were used to detect the cognitive memory function of AD mice after administration. Spatial memory may reflect only one cognitive function. Assessing the expression levels of Aβ plaques and synaptic-related proteins in the brain can provide a more quantitative assessment of the effect of treatment. The formation of (Aβ) plaques is a neuropathological indicator and a main feature of Alzheimer’s disease (AD) (Meadowcroft et al., 2009). Synaptic-associated proteins are important components involved in maintaining synaptic morphology and function. Their loss will lead to neurological diseases such as cognitive impairment (Johnson et al., 1992). SYN and PSD-95 are closely related to the degree of AD. In this study, our results indicated that Aβ protein expression in AD mice was upregulated, and Aβ protein expression was significantly reduced after WGA-NPs-miR132 treatment. Compared with the AD group, the expression of SYN and PSD-95 was significantly increased after WGA-NPs-miR132 treatment. In this study, it was also observed that the administration of WGA-NPs-miR132 had a significant protective effect on neurons because it could significantly inhibit the apoptosis of cells in the cerebral cortex and peripheral nerve cells.

To evaluate the effect of nasal delivery of the WGA-NPs-miR132 complex on cerebral ischemia-reperfusion injury in MCAO model rats, we selected several representative detection indicators. After an ischemic brain injury, microglial cells are activated to promote the release of inflammatory cytokines, and the inflammatory response is the main factor that leads to brain injury. It is well known that continuous activation of proinflammatory cytokines and enhanced expression of GFAP will aggravate brain damage, and inhibition of these cytokines and activated astrocytes is beneficial for the treatment of ischemic brain damage (Lee et al., 2006). Therefore, we chose the microglia marker Iba-1 and astrocyte marker GFAP to detect the effect of WGA-NPs-miR132 in the treatment of ischemic brain injury. Inflammatory reactions can lead to secondary brain damage, including cell death (Matsushita et al., 2012). We evaluated the effects of nasal inhalation and delivery of WGA-NPs-miR132 by evaluating apoptosis around brain lesions after cerebral ischemia. The area of cerebral infarction, neurological score, apoptotic ratio, and Western blot were used to evaluate the therapeutic effect of this treatment on ischemic brain injury. We found that treatment with the WGA-NPs and naked-mir132 were not significantly different from the model group, indicating that the carrier itself had no protective effect against ischemic brain damage. The nucleic acid delivered alone was easily cleared by the nasal cilia, and the nucleic acid was easily degraded, which prevents it from being inhaled into the brain through nasal aspiration. NPs-miR132 and WGA-NPs-miR132 reduced the area of cerebral infarction, the number of microglia was significantly reduced, and the number of apoptotic cells after cerebral hemorrhage was reduced; in total, the WGA-NPs-miR132 had a more significant therapeutic effect than any of the other groups. It was shown that nasal delivery of WGA-NPs-miR132 could be used as a highly efficient, safe and convenient method of administration with great potential for the treatment of ischemic stroke.

The above experimental results showed that the therapeutic effect of WGA-NPs-miR132 was significantly better than that of naked-miR132 or NPs-miR132 in experimental animals with cerebral ischemia and Alzheimer’s disease. It was shown that miRNA could have enhanced stability *in vivo* after being wrapped by nanoparticles, and it could avoid the degradation by enzymes such as P450 in the nasal mucus. Modifying nanoparticles with WGA reduced the chance of nasal ciliary clearance and enhanced their targeting to neurons.

## Conclusion

Nanocarriers are very effective in providing enhanced delivery of therapeutic drugs to the brain through an intranasal route. WGA-NPs-miR132 by nasal administration can enable to cross the BBB, which increasing drug bioavailability in the brain; thus, this strategy can be used to treat brain diseases. Therefore, nasal delivery of nanoparticle-encapsulated miRNA has great potential in the treatment of neurodegenerative diseases.

## Data Availability Statement

All datasets generated for this study are included in the article.

## Ethics Statement

The animal study was reviewed and approved by Animal Ethics Committee of the School of Pharmaceutical Sciences, Jilin University.

## Author Contributions

BH and YS contributed conception and design of the study. XD, LF, and YZ collected the samples for this study. YS, BS, and XG performed the experiments. ZL, YW, and HJ analyzed the data. YS wrote the first draft of the manuscript. BS and XG critically revised the manuscript. All authors contributed to the article and approved the submitted version.

## Funding

This work was funded by the Industrial Technology Research and Development Project form Jilin Province Development and Reform Commission (2019C050-3).

## Conflict of Interest

The authors declare that the research was conducted in the absence of any commercial or financial relationships that could be construed as a potential conflict of interest.
